# Antimicrobial Potential of Silver Nanoparticles Synthesized Using Medicinal Herb *Coptidis rhizome*

**DOI:** 10.3390/molecules23092268

**Published:** 2018-09-05

**Authors:** Garima Sharma, Ju-Suk Nam, Ashish Ranjan Sharma, Sang-Soo Lee

**Affiliations:** Institute for Skeletal Aging & Orthopedic Surgery, Hallym University-Chuncheon Sacred Heart Hospital, Chuncheon-si 24252, Gangwon-do, Korea; microbio.garima@gmail.com (G.S.); boneresearch@hallym.ac.kr (A.R.S.)

**Keywords:** silver nanoparticles, *Coptidis rhizome*, antibacterial, biosynthesis

## Abstract

*Coptidis rhizome* contains several alkaloids that are bioactive agents of therapeutic value. We propose an eco-friendly method to synthesize biocompatible silver nanoparticles (AgNPs) using the aqueous extract of *Coptidis rhizome*. Silver ions were reduced to AgNPs using the aqueous extract of *Coptidis rhizome*, indicating that *Coptidis rhizome* can be used for the biosynthesis of AgNPs. The time and the concentration required for conversion of silver ions into AgNPs was optimized using UV-absorbance spectroscopy and inductively coupled plasma spectroscopy (ICP). Biosynthesized AgNPs showed a distinct UV-Visible absorption peak at 420 nm. ICP analysis showed that the time required for the completion of biosynthesis was around 20 min. Microscopic images showed that nanoparticles synthesized were of spherical shape and the average diameter of biosynthesized AgNPs was less than 30 nm. XRD analysis also confirmed the size of AgNps and revealed their crystalline nature. The interaction of AgNPs with phytochemicals present in *Coptidis rhizome* extract was observed in FTIR analysis. The antimicrobial property of AgNPs was evaluated using turbidity measurements. *Coptidis rhizome*-mediated biosynthesized AgNPs showed significant anti-bacterial activities against *Escherichia coli* and *Staphylococcus aureus* that are commonly involved in various types of infections, indicating their potential as an effective anti-bacterial agent.

## 1. Introduction

Recently, nanotechnology has emerged as a promising interdisciplinary section dealing with research and development in various fields [1,2]. The advancements of nanotechnology-based approaches in various commercial segments and their direct effect on human life justify the need for their ecofriendly ways of synthesis [3]. Nanoparticles are generally described as small particles that measures around 1 nm to 100 nm in size [4]. The variations in shape, size, surface to volume ratio, and composition of nanosized metal nanoparticle provides them unique physical, chemical, and biological properties, that can be used in medicines, electronics, household devices, agriculture, cosmetics, and pharmaceutical areas [5,6,7,8,9,10].

Metal nanoparticles are synthesized using various approaches, such as biological, physical, or chemical methods with the conditions to control the size/shape and stability of nanoparticles [11]. The distinctive advantages of biological synthesis of nanoparticles, over physical and chemical synthesis methods, include the non-toxic nature of the process, simple process design, and cost effectiveness [12]. Biological methods of metal nanoparticle synthesis are performed through intracellular or extracellular approaches, generally utilizing plants and microorganisms. The potential applications of these biosynthesized nanoparticles are shown in a wide spectrum of areas, including cancer treatment targeted drug delivery, DNA analysis, gene therapy, antibacterial agents, magnetic resonance imaging (MRI), enhancing reaction rates, and biosensors [13].

Plant-mediated nanoparticle synthesis, also known as “green synthesis”, is the most widely acknowledged way of nanoparticle synthesis because of the plentiful and diversified cellular metabolites present in plant extracts [14]. These metabolites functions as bioactive compounds and are capable to act both as reducing and capping agents, thus eliminating the need to add any further chemical agent to synthesize the nanoparticles [14]. Recently, Vamanu et al. [15] showed the reduction of silver ions by phenolic compound homogentisic acid, which was present in the aqueous extract from *Lactarius piperatus* mushroom. The biodiversity of plants along with their phytochemical variations is the most accredited factor influencing the physical, chemical, and biological properties of biosynthesized nanoparticles as they generally get fabricated with nanoparticles during the synthesis progression.

Among metal nanoparticles, silver nanoparticles (AgNPs) are the most extensively studied nanomaterial. Silver has been used for ages as an excellent antimicrobial agent [16]. The conversion of silver into its nanoparticle form offers it unique physical and chemical properties that increases the efficacy of silver. AgNPs are used as disinfectant and as antimicrobial coating on medical instruments and devices, such as catheters [17,18]. Various mechanisms attributed to the antimicrobial activity shown by AgNPs, however, the exact mechanism is yet to be elucidated because the nanoparticles acts on different organisms in different ways. The development of antibiotic resistance by bacterial cells upon frequent use of antibiotics is a serious concern [19]. Recently, the promising antimicrobial potential of AgNPs against both gram positive and gram negative bacterial cells and their stability have attracted the scientific interest. Furthermore, by the use of AgNPs, the bacterial cells are less prone to develop antibacterial resistance [20].

*Coptidis rhizome* (*Coptis chinensis* Franch) has been known for its medicinal properties since ancient time. The dominant active compounds in aqueous extract of *Coptidis rhizome* include berberine, coptisine, jatrorrhizine, and palmatine. *Coptidis rhizome* has been well studied for its anti-diarrhea, anti-gastroenteritis, and chemotherapeutic properties [21,22]. However, the use of *Coptidis rhizome* for the biosynthesis of AgNPs is not reported. Here, we report *Coptidis rhizome* mediated eco-friendly ex-situ biosynthesis of AgNPs and the evaluation of their antimicrobial property against both Gram positive and Gram negative microorganisms.

## 2. Results

### 2.1. UV-Visible Absorbance of Coptidis rhizome-Mediated Biosynthesized Silver Nanoparticles (bAgNPs)

**Figure 1:**
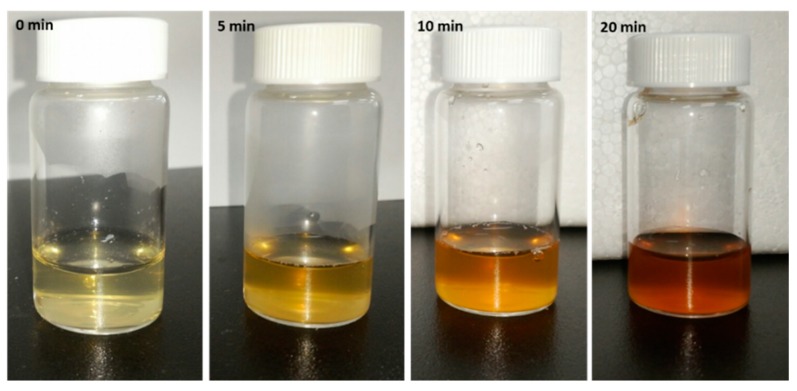
Time dependent variations in the colour of colloidal solution of silver nanoparticles synthesized using aqueous extract of *Coptidis rhizome*.

**Figure 2:**
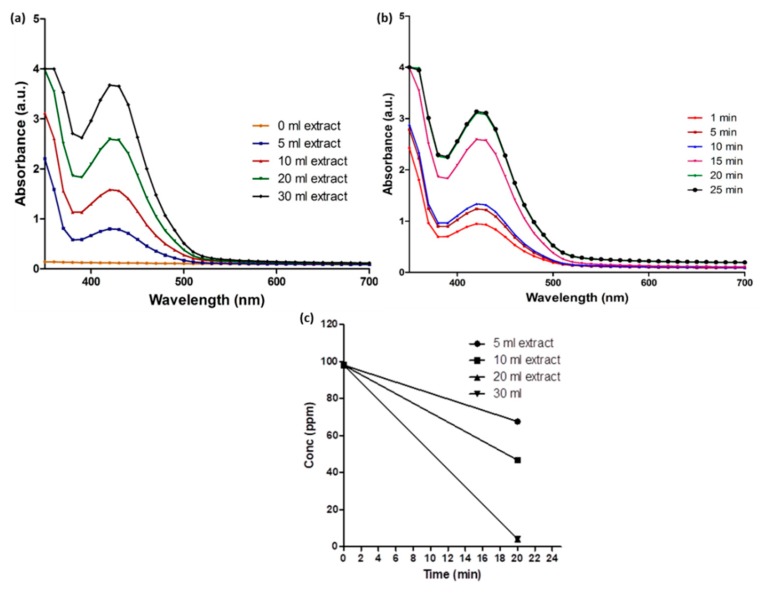
UV-Visible spectrum of *Coptidis rhizome*-mediated biosynthesized silver nanoparticles. (**a**) Surface plasmon resonance exhibited by biosynthesized silver nanoparticles using different concentration of *Coptidis rhizome*. (**b**) Surface plasmon resonance exhibited by biosynthesized silver nanoparticles at different time intervals. (**c**) Inductively coupled plasma graph of silver ions converted into zero valent form at different concentrations of *Coptidis rhizome*.

To observe the bioreduction potential of *Coptidis rhizome* extract and to optimize the required extract concentration for AgNPs synthesis, varying concentrations of plant extract (5 mL, 10 mL, 20 mL, and 30 mL) were added to 100 mL of AgNO_3_ solution and UV-Visible spectra of colloidal reaction solution was monitored. Soon after the addition of all the extracts, the color of AgNO_3_ solution changed from transparent to yellow and then to stable dark brown color within 20 min (Figure 1). This color change was supposed to be due to the reduction of AgNO_3_ into AgNPs. The surface plasmon resonance (SPR) spectrum of bAgNPs produced by using *Coptidis rhizome* showed a distinct absorption peak at 420 nm (Figure 2a), confirming the presence of AgNPs. The intensity of the peak was lowest in 5 mL extract concentration solution while, it was maximum in reaction solutions with 20 mL and 30 mL of extracts. According to UV-Visible spectra, biosynthesis of AgNPs started immediately after the addition of extract as indicated by peak at 420 nm wavelength within 1 min of extract addition. Further, the intensity of UV-Visible spectra peak became almost stable after 20 min of reaction time suggesting the completion of AgNPs synthesis (Figure 2b).

An absorption band at 280 nm observed in UV-Visible absorption spectra is plausibly attributed to electronic excitation in tryptophan and tyrosine residues in proteins [23]. This implies the presence of extracellular proteins in the colloidal solution and their possible mechanism in the bioreduction process [24].

### 2.2. Inductively Coupled Plasma (ICP) Spectroscopic Analysis of Coptidis rhizome-Mediated Biosynthesized Silver Nanoparticles (bAgNPs)

The estimated time and extract concentration required for maximum bioreduction of Ag ions into zero valent AgNPs was further confirmed using ICP. As shown in Figure 2c, 5 mL and 10 mL of the extract concentration decreased approximately 30.38% and 51.21% of Ag ions, respectively, within 20 min. However, 94% of Ag ions were reduced by 20 mL and 30 mL of extract concentrations within 20 min of reaction time. Therefore, the optimum extract concentration and time for maximum synthesis of AgNPs were estimated to be 20 mL and 20 min, respectively.

### 2.3. X-ray Diffraction (XRD) Analysis of Coptidis rhizome-Mediated Biosynthesized Silver Nanoparticles (bAgNPs)

**Figure 3:**
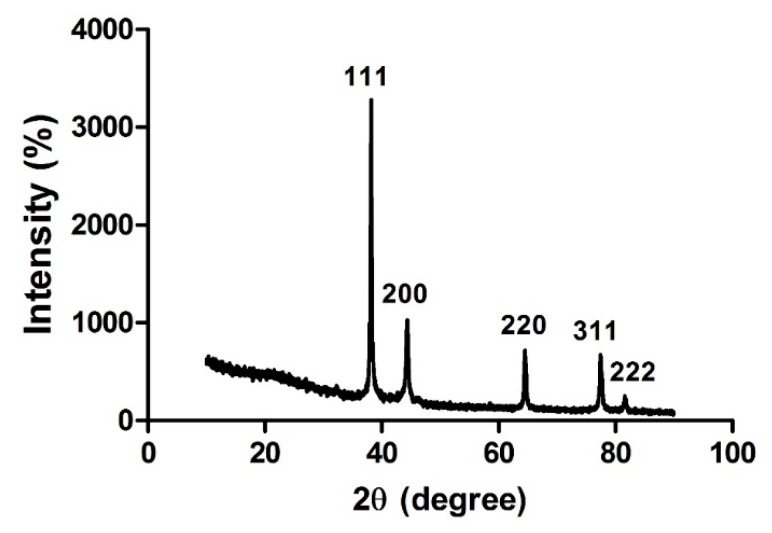
X-ray diffraction pattern of *Coptidis rhizome*-mediated biosynthesized silver nanoparticles.

XRD study was performed to identify the crystalline structure of bAgNPs. Five intense diffraction peaks were observed at 38.14, 44.31, 64.48, 77.42, and 81.55 angles (2θ degree) which correspond to 111, 200, 220, 311, and 222 Bragg’s reflection, respectively (Figure 3). These XRD peaks can be indexed as face-centered cubic silver (fcc), demonstrating high crystalline form of bAgNPs. These XRD peaks were also used to calculate the size of crystalline bAgNPs by Scherrer’s equation.

(1) D=0.94 λβcosθ

where D is the size of the nanoparticle, *λ* is the wavelength, *β* is the full width half maximum, and *θ* is the diffraction angle. According to different refraction peaks, the size of bAgNPs was calculated and is shown in Table 1. The results showed that average size of bAgNPs is 26.42 nm.

### 2.4. Transmission Electron Microscopic (TEM) Images of Coptidis rhizome-Mediated Biosynthesized Silver Nanoparticles (bAgNPs)

**Figure 4:**
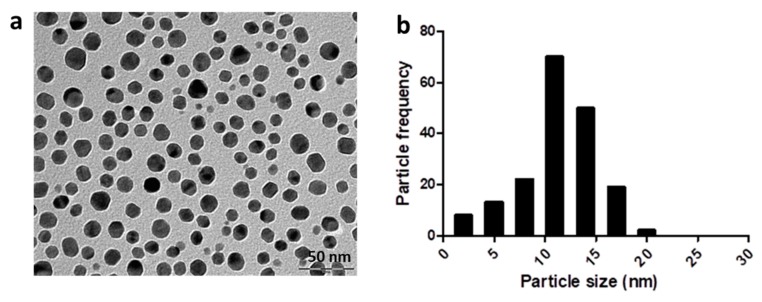
Transmission electron microscopic image of *Coptidis rhizome*-mediated biosynthesized silver nanoparticles. (**a**) Representative image. (**b**) Particle size histogram.

Additionally, the morphology and size of the bAgNPs were also examined by TEM. A representative TEM image showed that the *Coptidis rhizome* mediated bAgNPs were roughly spherical shape and evenly distributed in the sample (Figure 4). The observed nanoparticles in TEM image were in the range of 5 nm to 25 nm in diameter. 

### 2.5. Fourier Transform Infrared (FTIR) Spectrophotometric Analysis of Coptidis rhizome-Mediated Biosynthesized Silver Nanoparticles (bAgNPs)

**Figure 5:**
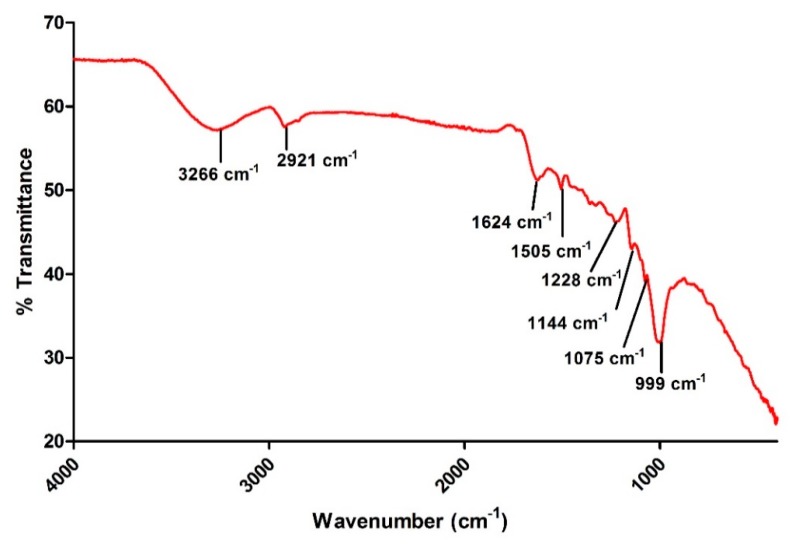
Fourier transform infrared spectrum of *Coptidis rhizome*-mediated biosynthesized silver nanoparticles.

The possible interaction of bAgNPs and the phytochemicals present in *Coptidis rhizome* extract was determined using FTIR. These phytochemicals were presumed to act as stabilizing and reducing agents. Figure 5 shows the FTIR spectrum in the range of 400 cm^−1^ to 4000 cm^−1^ demonstrating varying peaks located at 3266 cm^−1^, 2921 cm^−1^, 1624 cm^−1^, 1505 cm^−1^, 1228 cm^−1^, 1144 cm^−1^, 1075 cm^−1^, and 999 cm^−1^.

### 2.6. Antimicrobial Effect of Biosynthesized Silver Nanoparticles (bAgNPs)

**Figure 6:**
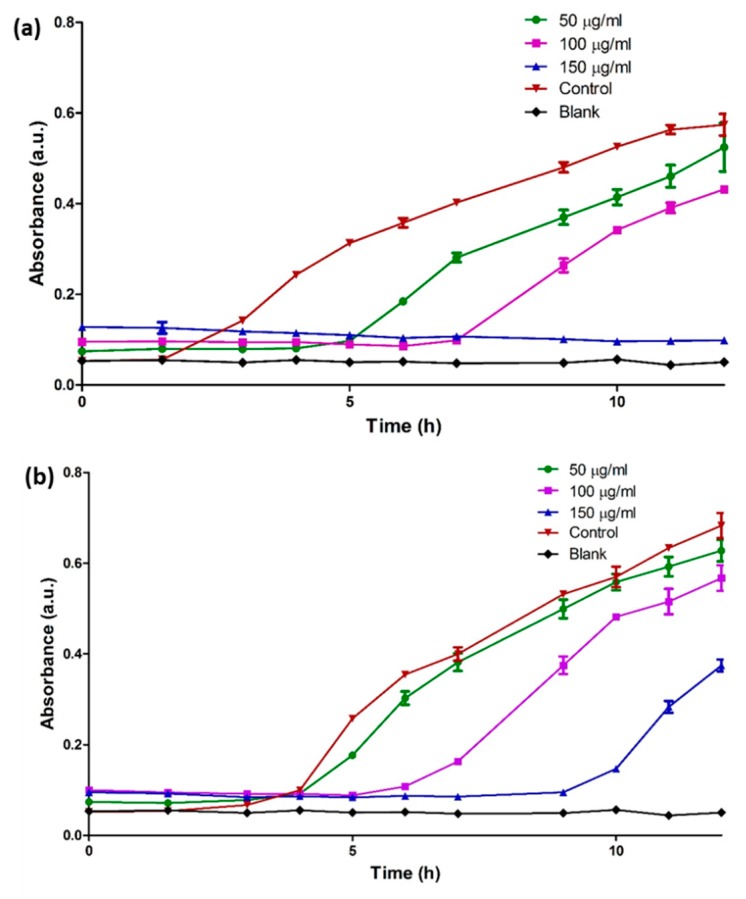
Antimicrobial study of *Coptidis rhizome*-mediated biosynthesized silver nanoparticles. (**a**) *Escherichia coli* (**b**) *Staphylococcus aureus*.

Here, turbidity measurements were used to evaluate the antimicrobial activity of bAgNPs against both Gram negative (*E. coli*) and Gram positive (*S. aureus*) microorganisms. Results showed dose dependent decrease in the bacterial growth upon treatment with bAgNPs. As compared to control, bAgNPs tend to increase the lag phase in bacterial growth curve of both the microorganisms at all the treated concentrations (50, 100, and 150 µg/mL) (Figure 6). It was observed that at high concentration (150 µg/mL), *Coptidis rhizome*-mediated bAgNPs completely inhibited the growth of *E. coli* until almost 12 h. Furthermore, the results also showed that the antimicrobial effect of bAgNPs was stronger against *E. coli* than to *S. aureus*.

## 3. Discussion

Nowadays, plant-assisted biosynthesis of metal nanoparticles has been extensively studied as an eco-friendly and efficient way to exploit biomolecules of plants as convenient bio-reducing agents [25]. In this study, within 20 min of the exposure of *Coptidis rhizome* extract, the color of AgNO_3_ solution changed from transparent to dark brown color which is a primary indication of AgNPs production (Figure 1). The color change was in agreement to previously reported studies that demonstrated the appearance of brown color due to excitation of surface plasmon vibrations with the synthesized AgNPs [26,27]. Furthermore, the production of bAgNPs was confirmed by UV-Visible spectroscopic analysis which quantifies the absorption spectra produced due to collective excitation of conduction electrons in silver (Figure 2). In this study, the UV-Visible spectra of colloidal reaction solution showed a sharp characteristic peak at around 420 nm wavelength which was produced due to SPR [28]. The optical absorption spectra of metallic nanoparticles are predominantly dominated by SPR and the peak of absorption correlated with particle size [29]. The shifting of the SPR peak of AgNPs in aqueous dispersion towards longer wavelengths is associated with the increase in particle size. Previous studies showed the effect of nanoparticle size, shape, and dielectric environment on the SPR band of noble metal nanoparticles [30,31]. Time dependent UV-Visible spectra analysis showed that maximum bioreduction was done within 20 min of reaction time which was further confirmed by ICP analysis (98%).

Although the exact mechanism of plant extract-mediated silver nanoparticle biosynthesis is not fully elucidated, some potential mechanisms have been proposed to explain the biosynthesis phenomenon [11]. It has been proposed that the presence of cellular enzymes in the plant extract can efficiently reduce silver ions into silver nanoparticles [11]. Besides reducing enzymes, other phytochemicals can also reduce silver ions into SNPs and can also act as capping agents to prevent nanoparticle aggregation [3,5].

The biosynthesis of crystalline AgNPs was confirmed by XRD spectrum which demonstrated peaks at 2*θ* of 38.14, 44.31, 64.48, 77.42, and 81.55 corresponding to 111, 200, 220, 311, and 222 face-centered cubic crystallographic planes of silver crystals (Figure 3). The results were in agreement to JCPDS card file no. 01-087-0717 [32]. The similar reflection peaks were reported in the *Ocimum sanctum*-mediated synthesized silver nanoparticles [14]. Additionally, the average size of bAgNPs was also calculated using Scherrer’s formula [33], which was 26.42 nm. Therefore, it can be assumed that phytochemicals present in the aqueous extract of *Coptidis rhizome* served as reducing agent during the AgNPs biosynthesis process.

Additionally, TEM images revealed the absence of aggregated nanoparticle clusters showing evenly distributed spherical bAgNPs in the sample (Figure 4). Thus, TEM images suggest that the phytochemicals present in *Coptidis rhizome* extract may serve as capping agents. The size of bAgNPs as shown in the TEM image was in the range of approximately 5 to 25 nm which was similar to the average size obtained by XRD analysis. The size of nanoparticles observed in TEM images was in accordance with previous reports [33,34]. Size and shape of nanoparticles are important factors affecting their biological properties [35].

The FT-IR spectra showed a broad absorption peak at 3266 cm^−1^ which may be attributed to hydroxyl (O–H) group of deionized water [36]. The presence of C–H stretching of aromatic compound was also ascertained by the absorption peak at 2921 cm^−1^ [37]. Additionally, the peaks at 1624 cm^−1^ and 1505 cm^−1^ are ascribed to –C=C– and =NH stretching of amide bonds, respectively [38]. This indicated that the amino groups are involved in the encapsulation and stabilization of bAgNPs. According to a previous study, absorption peak at 1505 cm^−1^ corresponds to flavonoids signifying the role of flavonoids in the bioreduction of silver ions into AgNPs [39]. Terpenoids associated ether linkages are also suggested in the *Coptidis rhizome*–mediated bAgNPs due to the presence of absorption peak at 1228 cm^−1^ [40]. Carbonyl (C–OH) stretching of proteins at 1075 cm^−1^ was also observed in FTIR spectra [41]. Henceforth, the FTIR peaks obtained here showed the presence of various phytochemicals, including flavonoids, terpenoids, and proteins, in the aqueous extract of *Coptidis rhizome*. Furthermore, FTIR indicates the multi-functionality of *Coptidis rhizome* in the bioreduction and stabilization of AgNPs (Figure 5). 

Here, turbidity measurement were carried out to evaluate the antimicrobial potential of biogenic AgNPs against *E. coli* (gram negative) and *S. aureus* (gram positive) bacterial cells. The results demonstrate extended lag phase which suggests dose dependent antibacterial effect of bAgNPs on both Gram positive and Gram negative microorganisms in consistence to previous studies [14]. Various mechanistic approaches detailing biochemical, physiological and morphological effect of AgNPs on bacterial cells were proposed in previous studies. Li et al., demonstrated that AgNPs may cause damage to *E. coli* cell membrane and interferes with the function of membrane enzymes eventually leading to cell death [42]. Generation of oxidative stress and production of reactive oxygen species (ROS), possibly due to inhibition of respiratory enzyme, was also attributed to bacterial cell death in some studies [43,44]. Electron spin resonance spectroscopy studies showed that the uncontrolled generation of free radicals from AgNPs can bind to membrane lipids rendering porosity to the cell membrane, leading to impaired membrane functions and cell death [45]. Preferential binding of AgNPs to phosphorous containing molecules, such as DNA, has also been revealed that may cause impaired DNA replication in the microbes [46]. 

Silver salts have been used as antimicrobial agents for decades. Monodispersed small sized spherical nanoparticles provide a higher surface area that enhances the antibacterial potential of silver. In fact, the size-dependent interaction of SNPs with pathogenic bacteria and viruses has been reported by many authors [47,48]. Aqueous extract of *Coptidis rhizome* is also well known for its antimicrobial effect, indicating the presence of biomolecules that possess antibacterial potential [49]. In line with previous study [50], it is possible that the capping biomolecules present on *Coptidis rhizome*-mediated bAgNPs might contribute to the antibacterial effect of nanoparticles. However, the exact mechanism of AgNPs-induced antibacterial effect is still unclear. 

## 4. Materials and Methods

### 4.1. Materials

The dried rhizome of *Coptidis rhizome* (*Coptis chinensis* Franch) was purchased from Hanyagin Co., Ltd. (Youngcheon, Korea) as an oriental medicine. Silver nitrate (AgNO_3_) used in this study was purchased from Sigma, Aldrich, St. Louis, MO, USA. 

### 4.2. Preparation of Plant Extract

Plant extract was prepared as previously described with modifications [14]. The collected plant rhizome was washed twice with distilled water and then air dried in dark at room temperature (RT). Afterwards, the plant part was grounded to powder using electric grinder. To prepare the extract, 10 g of the ground powder was added to 100 mL of distilled water at 60 °C under stirring for 60 min. The solution was then centrifuged at 12,000 rpm for 15 min in refrigerated centrifuge (Combi 514R, Hanil, Korea) to remove the debris as pellet. The plant extract was collected as supernatant and was stored at 4 °C for further use.

### 4.3. Biosynthesis of Silver Nanoparticles (AgNPs)

The *Coptidis rhizome* extract was used for the bioreduction of AgNO_3_ into AgNPs. Briefly, for biosynthesis of AgNPs, various concentrations of *Coptidis rhizome* aqueous extract was added to 1 mM of AgNO_3_ at 40 °C and the reaction mixture was further stirred till the reaction mixture exhibited a stable color change to dark brown. The biosynthesized silver nanoparticle (bAgNPs) containing solution was then centrifuged at 13,000 rpm for 30 min in a refrigerated centrifuge (Combi 514R, Hanil, Korea) to purify bAgNPs as pellet. The collected bAgNPs were washed thrice with deionized and double distilled water to remove the silver ions and the remains of unbounded phytochemicals present along with nanoparticles. Finally, the purified bAgNPs were lyophilized and stored at 4 °C as powder for further experiments [3].

### 4.4. UV-Visible Absorbance Spectroscopy

To examine the reduction of Ag ions into AgNPs, aliquots of the reaction solution were studied for the optical property of bAgNPs by measuring UV-Visible spectrum at a resolution of 10 nm from 200 nm to 800 nm at RT using UV-Visible spectroscopy (SpectraMax M2e Multi-Mode Microplate Reader, Molecular Devices, LLC, San Jose, CA, USA) [14].

### 4.5. Inductively Coupled Plasma (ICP) Spectroscopy

To confirm the conversion of Ag ions into zero valent AgNPs, the concentration of Ag ions in the reaction solution was measured using inductively coupled plasma spectroscopy (ICP-OES, ICAP 6300 duo thermo scientific). In brief, aliquots of reaction solution were withdrawn and centrifuged at 12,000 rpm for 15 min to separate non-ionic AgNPs as pellet. The Ag ions present in the supernatant were further quantified using ICP and the percent reduction of AgNO_3_ into AgNPs was calculated using following formula [51].

(2) $$\%\;\mathrm{conversion}=\left(\frac{Ci-Cf}{Ci}\right)\times 100$$

where, *Ci* and *Cf* are initial concentration and final concentration, respectively.

### 4.6. Transmission Electron Microscopy (TEM)

The morphology and size of bAgNPs was determined using high resolution transmission electron microscopy (TEM; JEM-2100F, JEOL, Tokyo, Japan). For this, bAgNPs were dispersed in double distilled water and sonicated for around 20 min. A drop of sonicated sample of bAgNPs was then loaded onto carbon coated copper grid. After evaporating the excess solvent, bAgNPs were visualized using TEM which was operated at accelerating voltage of 0–30 kV [14].

### 4.7. Fourier-Transform Infrared (FTIR) Spectroscopic Analysis

The vibrational bonding between the bAgNPs and phyto-molecules attached on their surface were analyzed using Fourier transform infrared spectrophotometer (FT-IR) with attenuated total reflectance (ATR) crystal (Perkin Elmer, Waltham, MA, USA). The FT-IR spectrum was recorded in the range of 400–4000 cm^−1^ with 4 cm^−1^ spectral resolution [14].

### 4.8. X-ray Diffraction (XRD) Analysis

XPERT-PRO diffractometer was used to perform X-ray diffraction (XRD) measurements of *Coptidis rhizome* mediated biologically synthesized AgNPs. In brief, AgNPs were casted onto glass slides and were analyzed in X-ray diffractometer operating at Cu-K(α) radiation of 1.5418 Å wavelength, 40 kV voltage, and 30 mA current. The scanning was done in the 2θ range from 10° to 90° [3].

### 4.9. Antimicrobial Studies

Antimicrobial activity of bAgNPs was determined as previously described by Okafor et al. [52]. The bAgNPs were prepared as previously described. To minimize the possibility of additive antimicrobial effect of *Coptidis rhizome* extract, nanoparticles were washed three times with D/W to remove extract and unbound biomolecules. For antimicrobial assays, the purified bAgNPs were first dispersed in autoclaved deionized water with the help of ultrasonicator to prepare a stock solution. Various concentrations of dispersed AgNPs were then prepared from the stock solution for the treatment. Microbial cultures (*Escherichia coli* ATCC 25922 and *Staphylococcus aureus* ATCC 29213) were grown in autoclaved nutrient broth medium which was prepared by adding NaCl (5 g/L), peptone (5 g/L), and beef extract (3 g/L) in deionized distilled water. Stock cultures of *Escherichia coli* and *Staphylococcus aureus* were then inoculated separately in the nutrient broth medium and incubated overnight. Afterwards, 15 µL of overnight bacterial cultures were added separately to each flask containing 15 mL of nutrient broth. The as-prepared microbial suspensions were treated with different concentrations of dispersed *Coptidis rhizome* mediated bAgNPs. Flasks were kept in incubator shaker at 150 rpm/min at 37 °C for 24 h. Blank nutrient broth was taken as control. At pre-determined time points, aliquots from all the treatment flasks were observed for bacterial growth using UV-Vis spectrophotometer at 600 nm wavelength. 

## 5. Conclusions

The present study concludes a successful and rapid biogenic process for AgNPs synthesis using aqueous extract of *Coptidis rhizome*. Phytochemicals present in the extract served as efficient bio-reducing and capping agents. Furthermore, this study also described the antimicrobial potential of biogenic AgNPs against bacterial cells, suggesting its application as an efficient antimicrobial agent in the future. However, the detailed mechanisms of antibacterial action are yet to be uncovered in future studies.

## Figures and Tables

**Table 1:** Average particle size of *Coptidis rhizome*-mediated biosynthesized AgNPs calculated using Scherrer’s equation.

Angle (2θ)	FWHM	Size
38.14	0.2747	29.6 nm
44.31	0.4471	19.1 nm
64.48	0.2736	35.5 nm
77.42	0.3946	25.8 nm
81.55	0.5220	22.1 nm

FWHM: full width and half maximum.
